# Critical care medicine: ICU survey in the Shandong Province in 2023

**DOI:** 10.1186/s13054-023-04523-5

**Published:** 2023-06-12

**Authors:** Xuan Song, Xinyan Liu, Wei Fang, Qizhi Wang, Jicheng Zhang, Zijian Tai, Chunting Wang

**Affiliations:** 1 ICU, Shandong Provincial Hospital Affiliated to Shandong First Medical University, 9677 Jingshi Road, Jinan, 250014 Shandong China; 2 Shandong Institute of Endocrine & Metabolic Diseases Affiliated to Shandong First Medical University, Jinan, 250062 Shandong China; 3 ICU, Dong E Hospital, Liaocheng, 252200 Shandong China

Dear Editor,

Intensive care unit (ICU) has played a huge role during the COVID-19 pandemic, and received unprecedented attention from the government and National Health Commission. ICU has been greatly developed after the pandemic. In 2015, we surveyed the structure and organization of ICUs in the Shandong Province, but these data no longer provide accurate support for government decision-making and better allocation of medical resources [1]. At present, there is no national ICU data in China, so we conducted a survey in Shandong Province again to understand the status of ICU development.

We used an online questionnaire to survey 755 ICU units in Shandong Province, from which we concluded the following points (Table 1): (1) The ratio of ICU beds to hospital beds is 3.3%, which is far behind developed countries such as Germany, Austria and the United States [2]. (2) The proportion of single rooms and negative pressure rooms is low. Although open rooms are more convenient for nursing and reduce staff demand, this design has a negative effect on infection control [3]. Given our experience with the COVID-19 pandemic, we believe that there should be more single rooms and negative pressure rooms. (3) The increase in convertible ICUs allows rapid conversion to a standard ICU within 24 h to meet the needs of a public health emergency. (4) The ratio of ICU doctors and nurses to beds is 0.52 and 1.50, respectively, far below the national standard. We are facing the dilemma of under-staffing. (5) Less than one in five ICUs perform daily multidisciplinary rounds (including respiratory therapists, rehabilitation therapists, and clinical pharmacists). Teamwork needs to be improved. (6) The extensive use of ICU information system and remote consultation systems has greatly alleviated the shortage of medical staff. Bronchoscopy, continuous renal replacement therapy (CRRT), ultrasound and other routine techniques have been widely implemented in all ICUs. Intra-aortic balloon pump (IABP) and extracorporeal membrane oxygenation (ECMO) are being progressively implemented.

**Table 1:** The characteristics of the surveyed ICUs

	ICUs in 2015	ICUs in 2023		
	All hospitals n = 305	All hospitals n = 755	Level II hospitals n = 337	Level III hospitals n = 418
Number of ICU beds	4084	12,209	3736	8473
Number of hospital beds	245,222	370,652	135,005	235,647
ICU beds/Hospital beds	1.7%	3.3%	2.8%	3.6%
Number of ICU physicians	2381	6362	2267	4095
Number Of ICU Nurses	7594	18,311	5793	12,518
ICU doctor/bed	0.6	0.5	0.6	0.5
ICU Nurse/bed	1.8	1.5	1.6	1.5
With respiratory therapists	24 (7.9%)	101 (13.4%)	27 (3.6%)	74 (9.8%)
With rehabilitation therapists	–	99 (13.1%)	31 (9.2%)	31 (9.2%)
With clinical pharmacists	–	125 (16.6%)	3 (10.1%)	91 (21.8%)
ICU information system	151(49.5%)	421 (55.8%)	157(46.6%)	264 (63.2%)
ICU remote consultation system	47 (15.4%)	145 (19.2%)	54 (16.0%)	91 (21.8%)
Bronchoscopy	175 (57.4%)	516 (68.3%)	293 (70.1%)	223 (66.2%)
CRRT	148 (48.5%)	427 (56.6%)	254 (60.8%)	173 (51.3%)
Bedside ultrasound	196 (64.3%)	496 (65.7%)	296 (70.8%)	200 (59.4%)
IABP	50 (16.4%)	140 (18.5%)	112 (26.8%)	28 (8.3%)
ECMO	33 (10.8%)	100 (13.3%)	91 (21.8%)	9 (2.7%)

*ICU* Intensive Care Unit, *CRRT* continuous renal replacement therapy, *IABP* intra-aortic ballon pump, *ECMO* extracorporeal membrane oxygenation

Since 2015, especially since the COVID-19 pandemic, ICU construction in the Shandong Province has improved significantly, but improvements must be made in terms of bed allocation and medical staff resources. In order to alleviate the dilemma, the government, national health commission and medical institutions have formulated a series of policies. Different from the pre-pandemic, these policies not only expand the ICU resources, but also change the management mode. In the future, the government will also introduce a series of policies to promote the development of ICU.

Expand and standardize ICU personnel training. Under the promotion of the government, we have established master's and doctorate degrees in critical care medicine, included critical care medicine in the Standardized Medical Residency Training and Specialty Training, and an ICU medicine an application code was granted by the National Natural Science Foundation of China. Providers can now specialize in ICU medicine, rather than transferring from emergency medicine, anesthesiology, or other specialties to the ICU without specialized ICU training. In addition, more people are paying attention to ICUs due to the important role they have played in the COVID-19 epidemic. The number of graduate students joining critical care medicine is significantly higher than before the COVID-19 pandemic, although we do not have exact data at present.

Homogenized management between comprehensive ICU and specialized ICU. Our survey found that there are more and more specialized ICUs. How to define the relationship between comprehensive ICU and specialized ICU is a problem that must be addressed in China. Firstly, staff in specialized ICU should be regularly trained on critical core diseases and core technologies to enable them to have comprehensive ICU ability. Second, comprehensive ICU physicians should be transferred to specialized ICU, and apply the concept of ICU to manage the patients in specialized ICU, so as to achieve specialization, standardization and homogenization of specialized ICU management. Finally, the establishment of ICU alliance system managed by the comprehensive ICU. This alliance system would allow ICUs of all kinds to share staff, equipment, and resources to achieve efficient operation.

Establish intelligent ICU based on informatization. In hospitals, early warning system and rapid response team (RRT) for critical patients have been established to provide immediate early warning and intervention for potential critical patients and transfer them to the ICU if necessary. Outside the hospital, relying on the central ICU and telemedicine, remote ward rounds and remote training are carried out to achieve homogenization. The emergency resource database should be established to reflect the distribution of medical resources in real time, including staff, beds and equipment, so as to realize the overall management of resources and provide information support for government decision-making.

## Data Availability

The datasets used and/or analyzed during the current study are available from the corresponding author on reasonable request.
